# Metabolites as Prognostic Markers for Metastatic Non-Small Cell Lung Cancer (NSCLC) Patients Treated with First-Line Platinum-Doublet Chemotherapy

**DOI:** 10.3390/cancers12071926

**Published:** 2020-07-16

**Authors:** Desirée Hao, Arjun Sengupta, Keyue Ding, ER Ubeydullah, Saikumari Krishnaiah, Natasha B. Leighl, Frances A. Shepherd, Lesley Seymour, Aalim Weljie

**Affiliations:** 1 Department of Medical Oncology, Tom Baker Cancer Centre and Cummings School of Medicine, University of Calgary, 1331-29th Street N.W., Calgary, AB T2N 4N2, Canada; 2 Department of Systems Pharmacology and Translational Therapeutics, Perelman School of Medicine at the University of Pennsylvania, Philadelphia PA 19104, USA; arjunsen@upenn.edu (A.S.); dr.ubeydullaher@gmail.com (E.U.); yksaikumari@gmail.com (S.K.); 3 Canadian Cancer Trials Group, Queen’s University, 10 Stuart Street, Kingston, ON K7L 3N6, Canada; kding@ctg.queensu.ca (K.D.); lseymour@ctg.queensu.ca (L.S.); 4 University Health Network, Princess Margaret Cancer Centre, 700 University Avenue, Toronto, ON M5G 1Z5, Canada; natasha.leighl@uhn.ca (N.B.L.); frances.shepherd@uhn.ca (F.A.S.)

**Keywords:** non-small-cell-lung cancer, metabolomics, lipidomics, NMR, UPLC-MS, overall survival

## Abstract

The metabolic requirements of metastatic non-small cell lung (mNSCLC) tumors from patients receiving first-line platinum-doublet chemotherapy are hypothesized to imprint a blood signature suitable for survival prediction. Pre-treatment samples prospectively collected at baseline from a randomized phase III trial were assayed using nuclear magnetic resonance (NMR) spectroscopy (*n* = 341) and ultra-high performance liquid chromatography – mass spectrometry (UPLC-MS) (*n* = 297). Distributions of time to event outcomes were estimated by Kaplan-Meier analysis, and baseline characteristics adjusted Cox regression modeling was used to correlate markers’ levels to time to event outcomes. Sixteen polar metabolites were significantly correlated with overall survival (OS) by univariate analysis (*p* < 0.025). Formate, 2-hydroxybutyrate, glycine and *myo*-inositol were selected for a multivariate model. The median OS was 6.6 months in the high-risk group compared to 11.4 months in the low risk group HR (Hazard Ratio) = 1.99, 95% C.I. (Confidence Interval) 1.45–2.68; *p* < 0.0001). Modeling of lipids by class (sphingolipids, acylcarnitines and lysophosphatidylcholines) revealed a median OS = 5.7 months vs. 11. 9 months for the high vs. low risk group. (HR: 2.23, 95% C.I. 1.55–3.20; *p* < 0.0001). These results demonstrate that metabolic profiles from pre-treatment samples may be useful to stratify clinical outcomes for mNSCLC patients receiving chemotherapy. Genomic and longitudinal measurements pre- and post-treatment may yield addition information to personalize treatment decisions further.

## 1. Introduction

Although targeted therapies and immunotherapy have reshaped the approach to treating metastatic non-small cell lung cancer (mNSCLC), chemotherapy remains an integral part of treatment algorithms for mNSCLC. Whether chemotherapy is used alone, or in combination with novel therapies, there is added toxicity from cytotoxic agents, thus predicting those who are most likely to benefit from the inclusion of chemotherapy remains important, even in the era of personalized medicine. Presently no prognostic or predictive biomarkers exist to identify which patients are most likely to benefit from chemotherapy. 

The current study was initiated as a proof-of-principal study to evaluate metabolomics as a platform for prognostic marker discovery. To date, studies of metabolic profiling in lung cancer patients have largely focused on its potential as a diagnostic tool [1]. Various metabolites have been found at higher levels in lung cancer tissues/patients versus controls/healthy volunteers, while others appear to distinguish between tumour subtype [2] and stage [3]. In our previous pilot study evaluating serum samples collected before, during, and after chemotherapy ± radiation, we have demonstrated that metabolite profiles varied temporally over the course of treatment [4]. Metabolite profiles have the potential to act as prognostic markers of clinical outcomes; metabolites such as hydroxylamine, tridecan-1-ol, octadecan-1-ol, were indicative of survival and metabolites such as tagatose, hydroxylamine, glucopyranose, and threonine that were reflective of progression. Building on this prior work, here we report the evaluation of metabolomic profiling in a larger, homogenous cohort of mNSCLC patients undergoing treatment with platinum-doublet chemotherapy with prospectively collected serum samples. Using a combination of nuclear magnetic resonance (NMR) and ultra-high performance liquid chromatography-mass spectrometry (UPLC-MS) lipidomics, we demonstrate that survival risk can be stratified based on the circulating levels of selected metabolites.

## 2. Results 

### 2.1. Patient Characteristics

**Figure 1:**
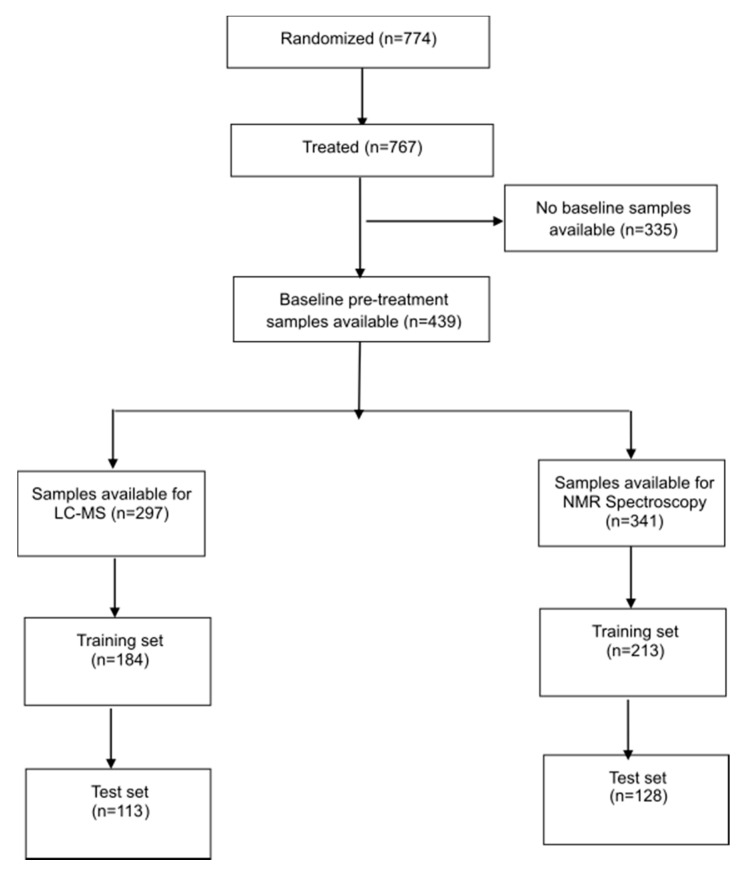
Study consort diagram describing the study population breakdown into training and validations sets for NMR spectroscopy and LC-MS.

Seven hundred and seventy four (774) patients from 63 participating centers in 14 countries were accrued to BR18 between April 2000 and May 2002. Full results of the clinical trial have been previously reported [5]. An interim safety analysis revealed no survival advantage for the experimental agent BMS-275291 vs. placebo groups (overall survival = 8.6 months vs. 9.2 months for BMS-275291 vs. control) leading to early termination of the study. The CONSORT diagram (Figure 1) describes the breakdown of the study population broken down into training and validations sets for NMR spectroscopy and LC-MS. 

Among the 774 patients recruited, 767 patients received treatment, of which 439 had baseline pretreatment serum samples collected. 341 and 297 serum samples were used for NMR spectroscopy and LC-MS respectively. Patients’ clinical characteristics by analytic method (NMR vs. LC-MS) are outlined in Table 1; Table 2. There were no significant differences in age, gender, performance status, treatment received or OS between patients with serum samples available for analysis and the overall study population. 

### 2.2. Overall Survival Analysis from NMR Data

NMR spectroscopy was used to quantitatively profile water soluble polar molecules of low molecular weight. Among the 341 samples in which NMR data was acquired, 213 were used as a training set and 128 in the test set. Concentration data of each metabolite was log_2_ transformed and z-scored by mean centering. Out of 43 polar metabolites, 16 were found to be significantly correlated with the overall survival rate using univariate cox regression (Table S1, *p* < 0.025). Final multivariate prognostic model was created from 4 metabolites (2-hydroxybutyrate, formate, glycine and *myo-*inositol) after backward variable elimination at 5% significance level (Table S1). 

**Figure 2:**
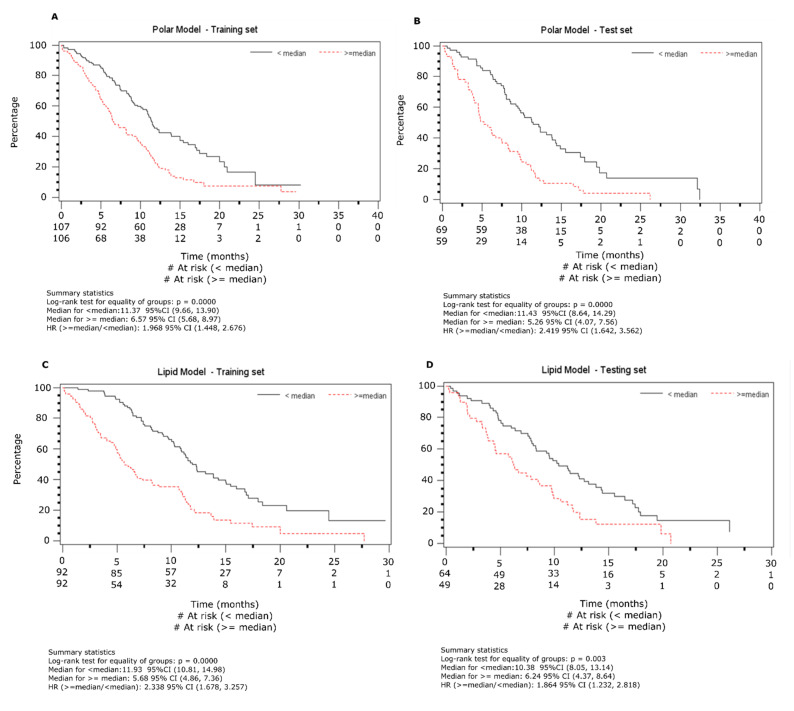
Prognostic survival analysis using the nuclear magnetic resonance (NMR) and lipid data sets. The data is presented using Kaplan-Meier curves on test and training sets from the two analytical platforms. Samples were divided into training and test sets as described in Figure 1. (**A**). NMR training dataset, (**B**). NMR test dataset, (**C**). Lipid training dataset and (**D**). Lipid test dataset.

Based on the model, each patient was assigned a prognostic score and were divided into two groups based on the median prognostic score. Median overall survival (OS) was found to be 11.4 months for the low risk and 6.6 months for the high-risk group (HR = 1.99, 95% C.I 1.45–2.68; *p* < 0.0001, Figure 2A). Multivariable Cox regression model adjusted for baseline factors was performed to check the high-risk group’s effect and found its effect is similar to the univariate analysis (HR: 2.16, 95% C.I. 1.57–2.98; *p* < 0.0001, Table 1).

The test set was used for validating the results found from the training set. In order to do so, the risk scores for patients in the test set were separated into two groups based on the median of the training set. The Kaplan-Meier (K-M) estimate of the survival distribution based on the median is presented in Figure 2B. The median OS are 11.6 and 5.3 months for the low and high-risk groups, respectively (HR = 2.43, 95% C.I 1.61–3.66; *p* < 0.0001). Multivariate Cox regression modeling, adjusted for baseline factors was performed to check the effect of the high risk group, and found to be similar to the univariate analysis (HR: 2.43, 95% C.I. 1.61–3.66; *p* < 0.0001, Table 1).

Among the four metabolites used for constructing the multivariable model, 2-hydroxybutyrate (2-HB, *p* = 0.001) and formate (*p* = 0.002) were found to be significantly associated to the OS in the test set. The correlation between two other metabolites, glycine (*p* = 0.11) and *myo*-inositol (*p* = 0.31), trended the same direction, but did not meet the significance threshold.

### 2.3. Overall Survival Analysis from Lipid Data

A total of 297 baseline lipid profiles were assayed. The dataset was randomly divided into two subsets with roughly 3:2 ratio (184 patients in training set and 113 in testing set). The data was log_2_ transformed and mean centered to construct z scores. A total of 1420 lipid features were analyzed via univariate Cox regression modeling to establish each variable’s correlation with OS from training set data and 53 variables (Table S2, *p* < 0.025) were identified. To take care of the inter-variable co-linearity due to biosynthetic pathways and other factors, these variables were subjected to hierarchical cluster analysis resulting in 12 branches (Table S3). For the branches with moderate to significantly correlated lipid features, principal component analysis (PCA) was carried out and the first principal component (PC) from each such branch was used as the representative variable for further analysis, 9 PCs were identified using this method. Backward variable elimination with a 5% significance level led final to a multivariable model using 4 variables — two of those include largely sphingolipids and triglycerides while specific lipid classes could not be assigned to the other two classes (Table S3). A prognostic score for each patient was generated as detailed in Supplementary Information S1. Based on these scores the patients were separated into high and low risk groups. Using Cox regression modeling, the median OS for the high-risk group was found to be 5.7 months while median OS for low risk group was 11.9 months (Figure 2C, HR: 2.34, 95% C.I. 1.68–3.26; *p* < 0.0001). Multivariate Cox regression modeling adjusted for baseline factors was performed and results were similar to the univariate analysis (Table 2, HR: 2.23, 95% C.I. 1.55–3.20; *p* < 0.0001). 

In the lipid data generated from LC-MS, four PC variables were used to create a risk score and patients were divided into high and low risk groups based on the median risk scores. The K-M estimate of the OS was found to be 10.4 and 6.2 months for the low and high-risk groups, respectively (Figure 2D, HR: 1.86, 95% C.I. 1.23–2.82; *p* = 0.003). As with the polar data, multivariable Cox regression modeling adjusted for baseline factors was performed to evaluate the effect of the high risk group; results were similar to the univariate analysis (Table 2, HR: 1.83, 95% C.I. 1.19–2.81; *p* = 0.006).

## 3. Discussion

Pre-treatment serum metabolites and lipid profiles in mNSCLC patients have the potential to serve as prognostic markers of clinical outcomes. Using prospectively collected serum samples from a large cohort of mNSCLC that was linked to outcomes, we demonstrated that with both polar data and lipid data, patients could be divided into high and low risk groups with differential OS. The results suggest that NMR and LC-MS characterize different, but complementary, metabolomic and lipidomic profiles that each identifies a high vs. low risk group even after correcting for known clinical prognostic variable such as gender, stage, histology and performance status. Tian et al. [6] recently reported analagous findings using pre-treatment serum metabolic profiles from a larger cohort of mNSCLC patients treated with platinum-doublet chemotherapy, providing support for this approach. The results from our study are complementary. The metabolite panel reported by Tian et al. was associated with longer median progression free survival, but was a different set of seven metabolites (hypotaurine, uridine, dodecanoylcarnitine, choline, dimethylglycine, niacinamide, L-palmitoylcarnitine) [6] perhaps reflecting differences in the patient population or the systemic therapy used.

**Figure 3:**
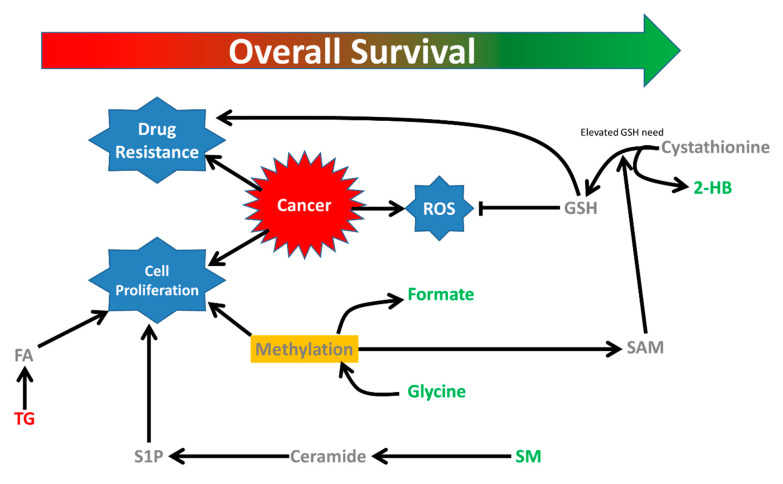
Metabolic changes associated with overall survival in NSCLC patients. Alteration in glutathione metabolic pathway is apparent from association of 2-hydroxybutyrate and survival. In addition, methylation pathways (formate, glycine), triglyceride and sphingomyelin metabolism are also associated to overall survival. FA: fatty acid, TG: triacylglycerol, S1P: Sphingosine-1-phosphate; SM: Sphingomyelin, SAM: S-adenosylmethionine, GSH: reduced glutathione, ROS: Reactive oxygen species, 2-HB: 2-hydroxybutyrate.

Results from our polar NMR data suggest that elevation of glutathione synthesis supported by elevated methylation pathways may be associated with better survival in NSCLC patients (Figure 3). 

We observed that elevated blood 2-HB, glycine and formate, were all positively associated with overall survival (Table S1). 2-HB is a metabolite related to glutathione synthesis [7]. Glutathione regulates the activity of ribonucleotide reductase that synthesizes deoxyribonucleotides from ribonucleotides [8] which is required for both cancer initiation and proliferation [9]. In NSCLC patients, elevated glutathione-S-transferase (GST) expression has been shown to decrease chemotherapeutic response [10,11]. The balance between the opposing effects of glutathione synthesis and metabolism may regulate the outcome of the disease. Glycine is also key component of glutathione synthesis [12].

Both glycine and formate are byproducts of transformylation via serine which donates the one carbon unit to tetrahydrofolate that further leads to purine/pyrimidine synthesis and transmethylation via the methionine cycle [13]. Two of the metabolites from Tian et al. are also involved in one-carbon metabolism (dimethylglycine, choline) [6]. Of note, another study designed to elucidate metabolites predictive for platinum-chemotherapy efficacy primarily identified caffeine-based metabolites [14] which affects plasma homocysteine level [15], a key reporter metabolite for one carbon metabolism. Hypermethylation facilitates cancer cell proliferation in various ways including reduced tumor suppressor gene expression [16], regulation of translation by methylation of RNA [17] and regulation of protein function by posttranslational modification [13].

Recent studies have shown that serine and glycine can provide the adenosine moiety needed for synthesis of S-adenosylmethionine (SAM) from methionine to support the methylation cycle [18] and in turn generate homocysteine which can lead to excessive systemic glutathione. The positive association of overall survival and methylation metabolites, glycine and formate, may ultimately be linked to glutathione metabolism. 

The lipid analysis of the patient serum samples provides further interesting insights to survival in NSCLC. Sphingolipids were positively associated to overall survival (Table S2). One of the three sphingolipids that was positively associated to overall survival was sphingomyelin (SM d18:2/25:0). The other two sphingolipids were putatively identified as ceramides based on their retention times. We hypothesize that elevated ceramide synthesis from membrane sphingomyelin may be associated to less aggressive tumorigenesis and better overall survival. Ceramide, a central sphingolipid which may be synthesized from sphingomyelin by sphingomyelinase [19], is associated with cell growth inhibition, induction of apoptosis, autophagy and ER stress response [20] and thus may be considered a tumor repressor lipid. We also observed several triglycerides were negatively associated to the overall survival. Cancer cells growth is enhanced in lipid-rich environments where the triglycerides are hydrolyzed to provide the fatty acids that may be used as the energy source by the cells [21]. Limiting fatty acids availability could serve as a potential therapeutic strategy against cancer [22], thus the negative association of triglyceride level with overall survival seen in this study reflects may reflect limited availability of fatty acids essential for the growth of cancer cells ultimately leading to improved survival of the patients. 

Other metabolomics studies in cancer patients have used different analytic platforms such as gas chromatography-mass spectrometry (GC-MS) and LC-MS. ^1^H-NMR can measure a wide range of metabolites with little sample preparation but is limited by a lower sensitivity and requires more expensive instrumentation. However, the technique is highly quantitative and hence the diagnostic value of biomarkers identified is much more useful from a clinical perspective. In contrast, hyphenated mass spectrometry generally requires more extensive sample preparation and can usually measure only a specific subset of metabolites depending on the type of hyphenation techniques that precedes the detection (GC/CE/LC). For example, hydrophilic interaction chromatography (HILIC) is more suitable for polar metabolites while reverse phase chromatography detects lipids/nonpolar metabolites more efficiently. The importance of polar metabolites in progression and prediction of survival in lung cancer have been underscored by different studies [14,23,24]. However, despite significant evidence of alteration in lipid metabolism in lung cancer [25], untargeted lipidomics analyses, as was performed in our study, have rarely been employed for biomarker discovery in lung cancer patients. NMR spectroscopy is not a suitable technique for measuring lipids and mass spectrometry coupled with reverse phase chromatography offers a more suitable approach. 

The main strength of our study was examined a large, homogenous population of patients with mNSCLC who were treated with first line cytotoxic chemotherapy. The serum samples we analyzed were uniformly collected prior to receiving systemic therapy hence the treatment would not have influenced our findings. Although patients in Canadian Clinical Trials Group (CCTG) BR18 study were treated with BMS-275291 vs placebo, the addition of BMS-275291 did not affect survival [5]; therefore our results serve as proof-of-principal that in mNSCLC patients receiving platinum-doublet chemotherapy, the pre-treatment metabolite profile could potentially be used as a prognostic marker. However, because the serum samples we evaluated were collected from a cohort dating back to 2000 to 2002, at a time when genomic profiling and/or tumour PDL-1 (Programmed death-ligand 1) status was not evaluated, it is conceivable that imbalances in tumour characteristics might have accounted for the observed differences between high and low risk groups. Confirmation in a more contemporary cohort would be necessary to substantiate these findings.

## 4. Methods and Materials

### 4.1. Patients

All serum samples used were prospectively collected from patients with mNSCLC participating in the CCTG BR 18 study, a randomized, double-blind phase III trial evaluating carboplatin, paclitaxel plus either placebo vs. the matrix metalloproteinase inhibitor BMS-275291 [5]. The samples were all collected and processed in a uniform fashion as per protocol. All samples were stored at the CCTG Tumour Tissue Data Repository in Kingston (Kingston ON, Canada). Clinical data including age, gender, stage, histology, and outcome were collected as part of the trial. This translational study was approved by the Health Research Ethics Committee of Alberta-Cancer Committee and the University of Pennsylvania.

As part of the original BR-18 trial, patients provided written informed consent with regards to participation in the trial and collection of blood samples for future studies.

This study was approved by the University of Calgary Conjoint Health Research Ethics Board (Ethics ID: E-24705, 2012-08-22) and the University of Pennsylvania Institutional Review Board (Protocol #816578, 2012-09-27). 

### 4.2. Sample Preparation 

We used two different methodologies to evaluate metabolomics signatures: nuclear magnetic resonance (^1^H-NMR) spectroscopy and liquid chromatography mass spectrometry (LC-MS). Blood serum (200 μL) was extracted using 1:2 chloroform/methanol. The upper polar fraction was dried using vacuum concentrator and the lower non-polar fraction was dried overnight in the hood. The polar fraction was further dissolved in 200 μL NMR solvent made up in 10% D_2_O with pH adjusted to ~7.0 by NaHPO4/NaH_2_PO4 and 0.25 mM 4,4-dimethyl-4-silapentane-1-sulfonic acid (DSS) added as internal standard. The reconstituted samples were put into 3 mm NMR tubes (Bruker Biospin, Billerica, MA, USA) in 96 tube racks designed for samplejet (Bruker Biospin) for acquiring the NMR spectra.

The non-polar fraction was used for analyzing the lipids. 40 μL of the fraction was dried and dissolved in 700 μL of solvent containing 60% solvent A (40% H2O, 60% ACN, 10 mM ammonium formate) and 40% solvent B (90% isopropanol 10% acetonitrile, 10 mM ammonium formate). 30 μL of the reconstituted solvent was added to microtubes containing 10 μL of internal standards (detail needed). The sample was further diluted to 150 μL using 60% A/40% B for injection. 

### 4.3. NMR Spectroscopy

All NMR spectra were acquired in a 700 MHz Bruker AVANCE III HD NMR spectrometer equipped with a 3 mm triple resonance inverse probe and Samplejet automated sample handling system (Bruker Biospin, Billerica, MA, USA). The pulseprogram used for acquiring the spectra took the shape of first transient of 2-dimensional NOESY spectroscopy (RD-90-t-90-t_m_-90-ACQ, where RD = relaxation delay, t = echo time, t_m_ = mixing time and ACQ = acquisition) [26]. Spectral acquisition was performed in Topspin 3.0 (Bruker Biospin). The FIDs (free induction decay) were acquired using 96 scans each acquiring 76K data points from a spectral width of 14 ppm with acquisition time 4 s for each FID. The FIDs generated was further exported for targeted NMR spectral profiling: 

Targeted spectral profiling [27] was performed using Chenomx v 8.0 (Chenomx Inc. Edmonton, AB, Canada). Imported FIDs were zero filled to 128k and applied line broadening factor 0.1 Hz and Fourier transformed. All spectra were calibrated with respect to the internal standard (0.25 mM DSS) and the spectral peaks were fitted with Chenomx reference library.

### 4.4. Lipidomics Assay by UPLC-Qtof-MS

Dried lipid fraction was reconstituted in 60% solvent A (40% H2O, 60% ACN, 10 mM ammonium formate) and 40% solvent B (90% IPA, 10% ACN, 10 mM ammonium formate). These samples were transferred into glass vials for ultra-performance liquid chromatography coupled with a qTOF Xevo G2S detector (Waters Corporation, Milford, MA, USA) for high throughput LC-MS-based lipidomics. 10 μL sample was injected into a reverse phase column (XSELECTTM CSHTM C18, 2.1 mm × 100 mm × 2.5 μm) using an Aquity H-class UPLC system (Waters Corporation). Samples were chromatographed for 9 min at 0.5 mL/min flow rate. The UPLC gradient was as follows - 75% A and 25% B for 0.5 min, a quick ramp of 50% A and 50% B for 0.5 min, 25% A and 75% B for 4 min, followed by a ramp of 10% A and 90% B for 2 min, and finally a ramp to 1% A and 99% B for 2 min. Column eluent was directly introduced into mass spectrometer that was performed in both positive and negative ion–sensitive mode with a capillary voltage of 3000 V and a sampling cone voltage of 40 °C. The desolvation gas flow was set to 800 L/h, and the temperature was set to 450 °C. The source temperature was set to 80 °C. Assessment of accurate mass was maintained by the introduction of a lock-spray interface of leucine-enkephalin (556.2771 m/z) at a concentration of 0.5 ng/μL in 50% aqueous acetonitrile and a rate of 5 μL/min. Data was acquired in the centroid MSe mode from 50–1200 m/z mass ranges for both MS (low energy) and MSe (high energy) modes. Low-energy or fragmented data were collected without collision energy, whereas high-energy or fragmented data were collected by using a collision-energy ramp from 15–40 eV. The entire set of duplicate sample injection was bracketed with test mix of standard metabolites at the beginning and end of run for evaluating instrument performance. Samples were randomized and injected in duplicates with pooled quality control sample injection after every 10 runs to compensate for instrumental drift. Data analysis and lipid identification were performed with Progenesis QI 2.3 software (Waters Corporation, Milford, MA, USA).

### 4.5. Statistical Analysis

Exploratory analyses were performed to characterize the relationships between patients’ lipid and polar metabolite levels with baseline characteristics and outcomes. Chi-square test was used to assess association between categorical variables; Principle component analysis was used to synthesize information of correlated variables. Kaplan-Meier curves were used to estimate the distributions of time to event outcomes, log-rank test was used to test difference between groups, and Cox regression model was used to correlate markers’ levels to time to event outcomes while adjusting baseline characteristics.

Prior to any analysis, patients with NMR and/or LC-MS data were randomly divided in approximately a 3:2 ratio into training and testing sets, stratified by treatment received (BMS275291 vs. Placebo), stage (III vs. IV), and ECOG PS (0, 1, vs. ≥2). Overall survival between the two cohorts were compared and found to be similar. We then used the 3/5 of the data as training set, and the remaining 2/5 of the data as test set.

## 5. Conclusions

In an era of rapidly changing standards of care that now include immunotherapy and targeted therapy, conventional cytotoxic chemotherapy still forms part of the treatment algorithm for most mNSCLC patients. Therefore, tools to guide clinicians regarding which patients are the most likely to benefit from the addition of chemotherapy remain relevant in current times. Our results align with other metabolomics studies and taken together, suggest that this minimally invasive strategy warrants further validation among mNSCLC. In addition, the evaluation of changes in metabolomics/lipidomic profiles over the course of treatment may provide further insights into which patients are most likely to benefit for a specific therapeutic approach.

## Figures and Tables

**Table 1:** Prognostic effects of polar metabolite markers on overall survival after adjusting for baseline factors.

**A. Polar Metabolites Training Set**			
Factors	Hazard ratio	95% C.I. of HR	*p*-Value
Risk group High Low	2.16 1	1.57–2.97	<0.0001
ECOG Performance status 2, 3 0, 1	1.47 1	0.89–2.41	0.13
Weight loss ≥5% <5%	1.89 1	1.35–2.66	0.0004
Sex Male Female	1.30 1	0.87–1.94	0.20
Disease Stage III IV	1.20 1	0.81–1.78	0.35
Histology Squamous Non-Squamous	0.99 1	0.70–1.42 1	0.98
Hemoglobin ^1^ Grade 1+ Grade 0	1.51 1	1.18–2.23	0.01
**B. Polar Metabolites Test Set**			
Factors	Hazard ratio	95% C.I. of HR	*p*-Value
Risk group High Low	2.42 1	1.61–3.65	<0.0001
ECOG Performance status 2, 3 0, 1	1.74 1	0.89–3.42	0.11
Weight loss ≥5% <5%	1.48 1	0.93–2.34	0.10
Sex Male Female	0.98 1	0.61–1.59	0.94
Disease Stage III IV	0.51 1	0.30–0.85	0.01
Histology Squamous Non-Squamous	0.97 1	0.59–1.59 1	0.90
Hemoglobin ^1^ Grade 1+ Grade 0	1.59	1.06–2.68	0.04

^1^: Within normal limits (grade 0), <Lower limit of normal (grade 1+). H.R.: Hazard ratio, C.I: confidence interval. ECOG Eastern Cooperative Oncology Group.

**Table 2:** Prognostic effects of lipid markers on overall survival after adjusting for baseline factors.

**A. Lipids Training Set**			
Factors	Hazard ratio	95% C.I. of HR	*p*-Value
Risk group High Low	2.23 1	1.55–3.22	<0.0001
ECOG Performance status 2, 3 0, 1	1.51 1	0.87–2.64	0.14
Weight loss ≥5% <5%	1.93 1	1.31–2.85	0.0009
Sex Male Female	1.62 1	1.02–2.56	0.04
Disease Stage III IV	1.20 1	0.80–1.81	0.38
Histology Squamous Non-Squamous	0.95 1	0.60–1.49 1	0.81
Hemoglobin ^1^ Grade 1+ Grade 0	1.52 1	1.12–2.34	0.03
**B. Lipids Test Set**			
Factors	Hazard ratio	95% C.I. of HR	*p*-Value
Risk group High Low	1.83 1	1.19–3.22	0.006
ECOG Performance status 2, 3 0, 1	1.70 1	0.75–3.86	0.21
Weight loss ≥5% <5%	1.55 1	0.86–2.79	0.15
Sex Male Female	1.11 1	0.61–2.02	0.83
Disease Stage III IV	0.58 1	0.32–1.08	0.09
Histology Squamous Non-Squamous	0.85 1	0.45–1.61	0.62
Hemoglobin ^1^ Grade 1+ Grade 0	1.60 1	1.03–2.68	0.04

^1^: Within normal limits (grade 0), <Lower limit of normal (grade 1+). H.R.: Hazard Ratio; C.I: Confidence Interval. ECOG Eastern Cooperative Oncology Group.

## Data Availability

The data is available at www.doi.org/10.5281/zenodo.3887012.

## Supplementary Materials

The following are available online at https://www.mdpi.com/2072-6694/12/7/1926/s1, Table S1: Polar metabolites that significantly correlated to overall survival in the training set measured by NMR, Table S2: Lipids that significantly correlated to overall survival in the training set measured by RP-UPLC-qTOF-MS, Table S3: 53 significant lipid features were subjected to hierarchical cluster analysis to compensate for the linearity due to chemical similarity, Supplementary information S1: Statistical methodologies for analyzing correlated lipid data.
