# Supplementary material for: Metabolites as Prognostic Markers for Metastatic Non-Small Cell Lung Cancer (NSCLC) Patients Treated with First-Line Platinum-Doublet Chemotherapy
Source: Cancers (Basel). 2020 Jul 16;12(7):1926. doi: 10.3390/cancers12071926 (PMC7409233; doi:10.3390/cancers12071926)
Supplement: Supplementary file 1 [file cancers-12-01926-s001.pdf]

Supplementary Materials

Metabolites as Prognostic Markers for Metastatic Non-Small Cell Lung Cancer (NSCLC) Patients Treated with First-Line Platinum-Doublet Chemotherapy

Table S1. Polar metabolites that significantly correlated to overall survival in the training set measured by NMR. Metabolites highlighted in red are used for constructing final multivariate prognostic model after backward variable elimination at 5% significance level.

Metabolite	Estimate	*p*-Value	Hazard Ratio	HR Lower CL	HR Upper CL
2-Aminobutyrate	0.25507	0.0018	1.291	1.1	1.515
2-Hydroxybutyrate	0.26531	0.0009	1.304	1.114	1.526
2-Hydroxyisovalerate	0.16968	0.0152	1.185	1.033	1.359
Acetate	0.18074	0.0089	1.198	1.046	1.372
Formate	0.27353	0.0006	1.315	1.125	1.537
Glutamate	0.21519	0.007	1.24	1.06	1.45
Glycerol	0.22371	0.0002	1.251	1.11	1.409
Glycine	0.28807	0.0005	1.334	1.133	1.57
Isobutyrate	0.20947	0.0061	1.233	1.062	1.432
Isoleucine	0.17945	0.0146	1.197	1.036	1.382
Lactate	0.26943	0.0006	1.309	1.123	1.527
Methanol	0.29229	0.0002	1.339	1.15	1.56
N-Acetylcysteine	0.1833	0.004	1.201	1.06	1.361
Pyruvate	0.22064	0.0036	1.247	1.075	1.446
Succinate	0.32073	<0.0001	1.378	1.185	1.602
myo-Inositol	0.17335	0.0218	1.189	1.026	1.379

Table S2: Lipids that significantly correlated to overall survival in the training set measured by RP-UPLC-qTOF-MS. The first column is formatted as retention time_m/z.

Parameter	Estimate	Prob ChiSq	Hazard Ratio	HR Lower CL	HR Upper CL
0.49_264.1960	0.28951	0.0003	1.336	1.143	1.56
0.62_316.2478	0.18899	0.0164	1.208	1.035	1.41
0.63_324.1370	0.24208	0.0016	1.274	1.096	1.48
1.12_468.3076	−0.26673	0.0029	0.766	0.643	0.913
1.24_568.3389	−0.19298	0.0179	0.824	0.703	0.967
1.34_519.3350	−0.2079	0.0124	0.812	0.69	0.956
1.47_546.3613	−0.21871	0.0096	0.804	0.681	0.948
1.87_523.3643	−0.21667	0.0078	0.805	0.686	0.944
3.54_718.5953	−0.22491	0.01	0.799	0.673	0.948
3.61_627.5336	−0.21647	0.0095	0.805	0.684	0.949
3.62_904.5869	−0.22496	0.0049	0.799	0.683	0.934
3.88_790.5756	−0.1998	0.0181	0.819	0.694	0.967
3.89_790.5687	−0.19395	0.0229	0.824	0.697	0.973
3.95_792.5806	−0.27441	0.001	0.76	0.645	0.896
3.96_830.5666	−0.29875	0.0002	0.742	0.633	0.869

4.0_807.5528	−0.25354	0.0028	0.776	0.657	0.916
4.0_891.5382	−0.20696	0.0166	0.813	0.686	0.963
4.4_792.5852	−0.20391	0.0174	0.816	0.689	0.965
4.5_831.5955	−0.20608	0.0147	0.814	0.69	0.96
4.10_768.5858	−0.22121	0.0052	0.802	0.686	0.936
4.11_790.5683	−0.21792	0.009	0.804	0.683	0.947
4.12_742.5852	−0.18386	0.0221	0.832	0.711	0.974
4.13_834.5817	−0.20918	0.0136	0.811	0.687	0.958
4.14_833.5909	−0.22498	0.0095	0.799	0.674	0.946
4.14_834.6177	−0.21131	0.0156	0.81	0.682	0.961
4.16_834.5845	−0.23697	0.0075	0.789	0.663	0.939
4.20_724.5277	−0.20225	0.0237	0.817	0.686	0.973
4.30_793.5921	-0.24304	0.0046	0.784	0.663	0.928
4.30_848.5511	−0.19822	0.0122	0.82	0.702	0.958
4.30_900.5634	−0.32375	0.0001	0.723	0.613	0.854
4.39_808.5858	−0.19392	0.0249	0.824	0.695	0.976
4.50_794.6001	−0.23865	0.0075	0.788	0.661	0.938
4.50_812.6178	−0.22692	0.0035	0.797	0.684	0.928
4.50_919.5718	−0.20628	0.0133	0.814	0.691	0.958
4.52_776.5731	−0.25936	0.0031	0.772	0.65	0.916
4.53_834.5919	−0.19422	0.0072	0.823	0.715	0.949
4.69_772.6170	−0.20771	0.0108	0.812	0.693	0.953
5.34_849.6757	0.21205	0.0093	1.236	1.054	1.45
5.68_725.5448	−0.2378	0.004	0.788	0.67	0.927
5.71_647.6212	0.23097	0.0129	1.26	1.05	1.511
5.75_709.5519	−0.22528	0.01	0.798	0.673	0.947
6.54_894.7518	−0.21103	0.0157	0.81	0.682	0.961
6.69_849.6898	−0.19507	0.018	0.823	0.7	0.967
6.72_870.7503	−0.26861	0.0028	0.764	0.641	0.911
6.72_896.7689	−0.23399	0.0091	0.791	0.664	0.944
6.73_876.7172	−0.21128	0.0126	0.81	0.686	0.956
6.73_901.7269	−0.24575	0.0043	0.782	0.661	0.926
6.73_914.7842	−0.19079	0.0207	0.826	0.703	0.971
6.73_966.8473	−0.20427	0.0174	0.815	0.689	0.965
6.86_878.7341	−0.22529	0.0122	0.798	0.669	0.952
6.93_599.5025	−0.21072	0.0194	0.81	0.679	0.967
6.93_982.8757	−0.2079	0.0196	0.812	0.682	0.967
7.50_631.5620	0.25386	0.003	1.289	1.09	1.524

Table S3: 53 significant lipid features were subjected to hierarchical cluster analysis to compensate for the linearity due to chemical similarity. Most of the branches could be assigned based on chemical composition of the lipids. PC: Phosphatidylcholine, LPC: Lysophosphatidylcholine, SM: Sphingomyelin. Branches highlighted in red are used for prognostic score calculation based on PCA analysis as detailed in methods section.

Branch	Retention time_m/z	Assigned Lipid Class
1		Acylcarnitines
	0.62_316.2478	
	0.63_324.1370	

2		Sphingolipids
	5.34_849.6757	
	5.71_647.6212	
	7.50_631.5620	
3		Triglycerides
	6.73_966.8473	
	6.73_901.7269	
	6.72_896.7689	
	6.54_894.7518	
	6.93_599.5025	
	6.93_982.8757	
	6.72_870.7503	
	6.86_878.7341	
	6.69_849.6898	
	6.73_876.7172	
	6.73_914.7842	
4		Phospholipids
	3.88_790.5756	
	3.95_792.5806	
	3.89_790.5687	
	4.52_776.5731	
	4.20_724.5277	
5		Phospholipids
	1.24_568.3389	
6		Phospholipids (SM and PC)
	4.12_742.5852	
	4.39_808.5858	
	4.69_772.6170	
7		Phospholipids (SM and LPC)
	1.34_519.3350	
	3.54_718.5953	
8		Phospholipids
	4.04 -792.5852	
	4.50_794.6001	
	4.05_831.5955	
	4.10_768.5858	
	4.11_790.5683	
	3.61_627.5336	
	3.62_904.5869	
	3.96_830.5666	
	4.00_807.5528	
	4.14_833.5909	
	4.14_834.6177	
	4.13_834.5817	
	4.16_834.5845	
	4.30_793.5921	
	4.30_848.5511	
	4.30_900.5634	
9		Phospholipids

	4.50_812.6178	
	4.50_919.5718	
	4.00_891.5382	
	4.53_834.5919	
10		Unidentified
	5.68_725.5448	
	5.75_709.5519	
11		Phispholipids (LPC)
	1.12_468.3076	
	1.47_546.3613	
	1.87_523.3643	
12		Sphingosine
	0.49_264.1960	

Supplementary information S1: Statistical methodologies for analyzing correlated lipid data.

The multi-lipid markers model was developed based on training set. For the 53 variables that are selected for the multi-marker model, those variables start with the same number are highly correlated. According to certain criteria and the biotransformation pathway, those variables are classified into different branches using HCA (Table S2).

Branch 1; Acylcarnitine: 2 variables are not highly correlated, included as independent variables.

Branch 2, Sphingolipids: Moderately correlated variables with coefficients around 0.4 to 0.5. The first principle component (PC) explain 62.5% of variationthat was used for further analysis.

Branch 3, Triglycerides: Variables are highly correlated, the first PC explain 76% of variation.

Branch 4, Phospholipids: They are highly correlated, first PC explains 81% variation

Branch 5, consists of a single variable.

Branch 6, Phospholipids (1 SM, 2 PCs): highly correlated variables, and the first PC explains 82% variation.

Branch 7: Highly correlated variables and the first PC explains 90% variation.

Branch 8: Phospholipids: they are highly correlated, and the first PC explains 71% variation.

Branch 9: Phospholipids: The variables are highly correlated, and the first PC explains 81% variation.

Branch 10: The variables are highly correlated, and the first PC explains 81.5% variation.

Branch 11: The variables are moderately correlated, with correlation coefficients around 0.4 to 0.6, the first PC explains 65% variation.

Branch 12 includes a single variable.

(Note: It should be pointed out that, for those with multiple variables with similar association and highly correlated with each other, if a few are missed, and analysis based on available variables should give similar results.)

To keep the stable effect, branches that have multiple variables that correlated to OS for further multivariable prognostic model were used. Multivariable Cox regression model including variables of B21, B31, B41, B61, B71, B81, B91, B101 and B111 (B21 = 1st PC of variables from branch 2 from supplementary information S1), using backward variable elimination with 5% significance level were used for variable to stay, lead to the multivariable model with variables of: B21, B31, B101 and B111.

Based on the selected model, the prognostic score for each patient, i.e., sum of coefficients of those variables in the model multiply by the value of those variables (p = 0.25 *B21 -0.10*B31 - 0.23*B101 - 0.17*B111) were generated. Based on the median score, the population was divided into high and low risk population. Cox regression model with risk group indicator(s) was used to estimate the risk group(s)' effect.
